# Three-Dimensional Speckle-Tracking Echocardiography-Derived Aortic Valve Annular Plane Systolic Excursion and Left Ventricular Volumes Are Not Associated in Healthy Adults (Insights from MAGYAR-Healthy Study)

**DOI:** 10.3390/jcm15041507

**Published:** 2026-02-14

**Authors:** Attila Nemes, Nóra Ambrus, Tamás Szili-Török, Zoltán Ruzsa, Máté Vámos, Gábor Zoltán Duray, Csaba Lengyel

**Affiliations:** 1 Department of Medicine, Albert Szent-Györgyi Medical School, University of Szeged, 6725 Szeged, Hungary; ambrusnora@gmail.com (N.A.); szili-torok.tamas@med.u-szeged.hu (T.S.-T.); ruzsa.zoltan@med.u-szeged.hu (Z.R.); vamos.mate@med.u-szeged.hu (M.V.); lengyel.csaba@med.u-szeged.hu (C.L.); 2 Central Hospital of Northern Pest-Military Hospital, 1134 Budapest, Hungary; duray.gabor@epc-honvedkorhaz.hu

**Keywords:** three-dimensional, speckle-tracking, echocardiography, aortic valve, annulus, displacement, healthy, left ventricular volume

## Abstract

**Introduction**: The left ventricular (LV) function can be characterized by the spatial displacement of the aortic valve annulus (AVA), represented by the AVA plane systolic excursion (AAPSE). AAPSE, measured using 3DSTE, has been shown to be a complex feature of LV function, reflecting not only longitudinal function. The present study aimed to evaluate the potential associations between AAPSE and LV volumes as assessed simultaneously using 3DSTE in the same healthy adult individuals. It was also examined when AAPSE and LV volumes were average, or they were smaller or larger than average. **Methods**: The present study included 109 healthy subjects (mean age: 35.1 ± 12.0 years; 68 men). All participants underwent two-dimensional Doppler echocardiography, and 3DSTE was performed for the simultaneous evaluation of LV volumes and AAPSE. **Results**: No significant changes in LV volumes and LV-EF were observed in association with increasing AAPSE. LV-ESV and LV mass increased significantly with increasing LV-EDV, while LV-EF remained preserved. No significant changes in AAPSE could be detected with increasing LV-EDV. LV-EDV and LV mass also increased significantly with increasing LV-ESV. LV-EF proved to be significantly increased in the presence of the lowest LV-ESV. No significant changes in AAPSE were observed with increasing LV-ESV. No correlations were found between AAPSE and LV-EDV or LV-ESV. **Conclusions**: LV volumes and the spatial excursion of the AVA, represented by AAPSE, are not associated in healthy adults when measured simultaneously using 3DSTE.

## 1. Introduction

The left ventricular (LV) function can be characterized by the spatial displacement of the aortic valve (AV), represented by the AV annular (AVA) plane systolic excursion (AAPSE) [1,2,3,4,5]. According to the classic method, AAPSE is measured during M-mode echocardiography (MME) [1,2,3,4,5], but the usefulness of three-dimensional (3D) speckle-tracking echocardiography (3DSTE) has also been demonstrated in simultaneously measured AAPSE and LV volumes and strains [6,7,8]. It has been proven that AAPSE, as assessed through 3DSTE, is a complex feature of LV function, and not only of its longitudinal function [9]. Despite these findings, it is not known whether LV volumetric parameters respecting the cardiac cycle show a relationship with AAPSE, even in healthy individuals. Although it is important to understand the physiological relationships, these results should not be extrapolated to certain cardiovascular abnormalities or disorders with volume overload. Therefore, the present study aimed to assess the potential associations between AAPSE and LV volumes as evaluated simultaneously using 3DSTE in the same healthy adult individuals. It was also examined when AAPSE and LV volumes were average, or they were smaller or larger than average.

## 2. Subjects and Methods

**Subject population**: The study cohort consisted of 109 healthy individuals (mean age: 35.1 ± 12.0 years; 68 males). They volunteered to participate in the study in response to a recruitment call between 2011 and 2017**.** All clinical parameters—including physical examination, standard 12-lead electrocardiography (ECG), laboratory tests, and two-dimensional (2D) echocardiography with Doppler—were within normal reference ranges. Participants were selected based on the absence of known medical conditions or pathological states that might bias the findings. Exclusion criteria included regular medication use, smoking, pregnancy, professional athlete status, and obesity (body mass index > 30 kg/m^2^). The investigation forms part of the “**M**otion **A**nalysis of the heart and **G**reat vessels b**Y** three-dimension**A**l speckle-t**R**acking echocardiography in **Healthy** subjects” (**MAGYAR-Healthy Study**), which aims to explore the physiological relationships between 3DSTE-derived parameters and other clinical measures to enhance the understanding of cardiac mechanics in healthy individuals (“Magyar” means “Hungarian” in the Hungarian language). The study was conducted in accordance with the Declaration of Helsinki (revised 2013). Approval was obtained from the Institutional and Regional Human Biomedical Research Committee of the University of Szeged, Hungary (registration number: 71/2011; latest approval: 17 March 2025). Written informed consent was obtained from all participants.

**Two-dimensional (2D) echocardiography with Doppler:** 2D echocardiography with Doppler was performed in all cases to assess left atrial and LV dimensions, as well as LV ejection fraction (EF), calculated using the modified Simpson’s method [10]. Measurements were obtained using a Toshiba Artida^®^ cardiac ultrasound system (Toshiba Medical Systems, Tokyo, Japan) equipped with a broadband (1–5 MHz) PST-30BT phased-array transducer. Doppler echocardiography was also used to exclude significant valvular stenosis or regurgitation and to measure transmitral diastolic flow velocities—including early (E) and late (A) filling ones—and to calculate the E/A ratio.

**Three-dimensional speckle-tracking echocardiography:** 3DSTE was performed in two phases [11,12,13,14,15]. In the first phase, 3D echocardiographic datasets were acquired using the same Toshiba Artida^®^ cardiac ultrasound system, equipped with the 3D-capable PST-25SX matrix-array transducer. After optimizing imaging parameters (including magnitude, gain, and others), 3D datasets were acquired from the LV apical window. Participants were instructed to hold their breath to ensure optimal image quality, during which six subvolumes were captured over six consecutive cardiac cycles at stable RR intervals on ECG. The software automatically merged these subvolumes into complete 3D datasets.

**Figure 1:**
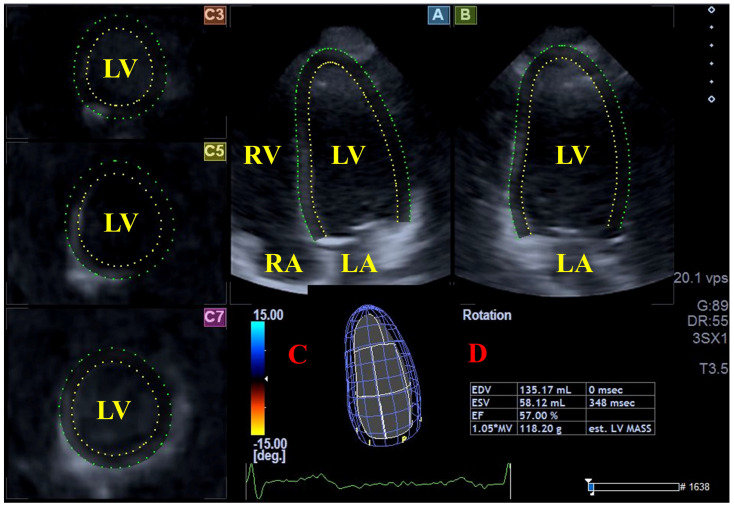
Three-dimensional (3D) speckle-tracking assessment of left ventricular (LV) volumes. From the acquired 3D echocardiographic datasets, the software automatically creates apical four-chamber (**A**) and two-chamber (**B**) long-axis views, along with short-axis views at the apical (C3), mid-ventricular (C5), and basal (C7) LV levels. These views allow for the generation of a 3D LV model (**Panel C**), which is then used to calculate precise LV volumetric parameters and the ejection fraction (**Panel D**). **Abbreviations:** LV = left ventricle, LA = left atrium, RV = right ventricle, RA = right atrium, EDV = end-diastolic LV volume, ESV = end-systolic LV volume, EF = LV ejection fraction.

The second phase, performed subsequently, involved analysis of the acquired datasets using the vendor-provided 3D Wall Motion Tracking software, version 2.7 (Toshiba Medical Systems, Tokyo, Japan). LV volumes and AAPSE were measured using the same 3D datasets. Based on apical two-chamber (AP2CH) and four-chamber (AP4CH) long-axis views, as well as three short-axis views at different LV levels, the observer placed markers on the apical LV endocardium and the mitral annular edges after appropriate optimizations. The software then reconstructed the LV endocardial surface and generated a 3D virtual LV cast following a sequential analysis. The following LV parameters were determined: end-diastolic and end-systolic LV volumes (LV-EDV and LV-ESV, respectively), LV-EF and LV mass (Figure 1) [16,17,18].

**Figure 2:**
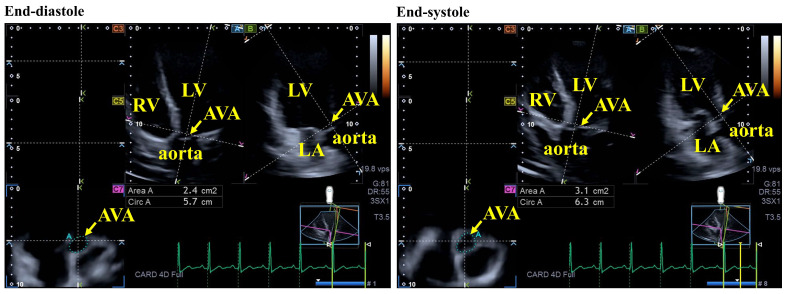
Evaluation of end-diastolic and end-systolic aortic valve annular (AVA) dimensions in apical four-chamber (A) and two-chamber (B) long-axis views by three-dimensional speckle-tracking echocardiography. ‘En-face’ view of the AVA is also presented in C7 cross-sectional view. Spatial AVA displacement is quantified by AVA plane systolic excursion. **Abbreviations:** AVA = aortic valve annulus, Area A = AVA area, Circ A = AVA perimeter, LA = left atrium, LV = left ventricle, RV = right ventricle.

To determine the position of the AVA, AP2CH and AP4CH long-axis views were used to obtain optimal LV longitudinal planes. After visualizing the AV and the aorta by appropriately tilting and optimizing these planes, the longitudinal views were aligned to be parallel to the centerline of the aortic root. The cross-sectional C7 view—used as the reference for AVA positioning—was then oriented perpendicular to these longitudinal planes. During measurements, particular care was taken to ensure that the C7 plane was truly perpendicular to the centerline and that assessments were not performed at the level of the sinus of Valsalva or the LV outflow tract. AAPSE was defined as the spatial displacement of the AVA from end-diastole to end-systole throughout the cardiac cycle (Figure 2) [6,7,8,19].

**Statistical analysis:** Data are presented as mean ± standard deviation (SD) or as counts with percentages. Homogeneity of variances was assessed using Levene’s test, while the Shapiro–Wilk test was applied to evaluate normality of distribution. For normally distributed variables, independent-samples *t*-tests were performed; for non-normally distributed variables, the Mann–Whitney–Wilcoxon test was used. When multiple comparisons were required, one-way analysis of variance (ANOVA) with Bonferroni correction was applied. For correlation analyses, Pearson correlation coefficients were calculated, and multivariable regression analysis has been performed. To evaluate the reproducibility of 3DSTE-derived measurements of LV-EDV, LV-ESV and AAPSE, the mean ± SD differences in values obtained twice by the same observer (intraobserver agreement) and by two independent observers (interobserver agreement) were assessed in 30 healthy individuals, together with their respective intraclass correlation coefficients (ICCs). A *p*-value < 0.05 was considered statistically significant. All statistical analyses were conducted using SPSS software (version 29.0.0.0, SPSS Inc., Chicago, IL, USA).

## 3. Results

**Clinical, 2D echocardiographic data with Doppler and 3DSTE data:** Data are summarized in Table 1. None of the participants exhibited a valvular regurgitation of grade ≥ 1 or clinically significant stenosis in any cardiac valve.

**Classification of subjects:** Healthy adults were categorized based on the mean ± standard deviation (SD) of 3DSTE-derived parameters: AAPSE (1.16 ± 0.30 cm), LV end-diastolic volume (LV-EDV, 86.9 ± 20.8 mL), and LV end-systolic volume (LV-ESV, 36.7 ± 10.6 mL). For the subgroup analysis, the following cut-off values were applied: AAPSE, 0.86 cm and 1.46 cm; LV-EDV, 66.1 mL and 107.7 mL; and LV-ESV, 26.1 mL and 47.3 mL.

**LV volumes in different AAPSE subgroups:** No significant changes in LV volumes or LV-EF could be detected in association with increasing AAPSE (Table 2). 

**AAPSE in LV-EDV subgroups**: LV-ESV and LV mass increased significantly with increasing LV-EDV, while LV-EF remained preserved. No significant changes in AAPSE could be detected with the increase in LV-EDV (Table 3).

**AAPSE in LV-ESV subgroups**: LV-EDV and LV mass increased significantly with increasing LV-ESV. LV-EF proved to be significantly increased in the presence of the lowest LV-ESV. No significant changes in AAPSE were detected with increasing LV-ESV (Table 4).

**Correlation and multivariable regression analyses:** No correlations could be detected between AAPSE and LV-EDV or LV-ESV. After adjusting for key covariates such as age, sex, weight, height, systolic and diastolic blood pressures, AAPSE, indexed LV volumes, and LV mass, the results remained consistent with the initial findings, suggesting that the lack of association was not driven by these confounding factors.

**Intraobserver and interobserver agreements:** The mean ± 2 SD differences in values obtained twice by the same observer and by two independent observers for the measurement of LV-EDV, LV-ESV and AAPSE using 3DSTE simultaneously, together with the corresponding ICCs, are demonstrated in Table 5.

## 4. Discussion

3DSTE seems to be suitable not only for the accurate measurement of LV volumes during the cardiac cycle [16,17,18], but, at the same time, using a single acquired 3D echocardiographic dataset, the spatial 3D displacement of the AVA represented by the AAPSE can also be measured [6,7,8,19]. 3DSTE is validated for LV volumetric assessment with well-defined normal reference values [16,17,18]. Based on the literature data, MME-measured AAPSE has been considered a useful parameter to characterize LV longitudinal function [1,2,3,4,5]. Nevertheless, its measurement is not widespread in clinical practice compared to similar parameters used to characterize mitral (MAPSE) and tricuspid (TAPSE) annular function [20]. A recent study from the MAGYAR-Healthy Study raised the possibility that 3DSTE-derived AAPSE is a more complex 3D functional parameter that shows some associations with all unidirectional, primarily apical regional LV strains. In addition, the inter- and intraobserver variability also proved to be adequate [9]. These initial findings may indicate the importance of the clinical utility of the 3DSTE-derived AAPSE, although further independent studies are needed, even in the form of measurements in certain pathological conditions. Moreover, 3D echocardiography has been demonstrated to be a useful technique for AVA assessments with 3DSTE-derived normal references as well [19,21]. Based on these facts, the question may rightly arise whether there is a detectable relationship between LV volume changes during the cardiac cycle and AAPSE.

Based on the results presented above, several facts and implications can be established. Firstly, it was demonstrated that 3DSTE is suitable for the simultaneous determination of LV volumes and AAPSE using a single acquired 3D dataset offline. This is important because it highlights the importance of 3DSTE for the direct investigation of physiological processes, even in healthy subjects, as demonstrated in the present study. Secondly, no associations were found between AAPSE and LV volumes. No correlations were detected either. This finding, considered as an extension of previous research, is both novel and interesting because, although the 3D pattern of the wall contractility showed a specific relationship with AAPSE, this relationship was not detectable through the LV volume change itself [9]. Third, it is very important to emphasize that the above presented findings were found in healthy adults, who were mostly younger in age. The absence of disease may itself explain the lack of association between AAPSE and LV volumes. These findings cannot be extrapolated to patients with heart failure, valvular disease, cardiomyopathy, or volume overload states. Elevated LV volumes are known to occur in many disorders or pathological states, and are often associated with abnormalities of quantitative features (strains) of LV contractility. From the presented findings, it may be inferred that changes in LV strains, rather than LV volume changes, show associations with AAPSE. In other words, the findings suggest that strain-based myocardial mechanics, rather than volumetric changes, may explain the behavior of AAPSE. Although scientifically plausible, this conclusion is indirect and was not formally tested in the study. Therefore, further studies would be necessary to confirm this. Moreover, in light of these findings, it is recommended that future studies extend these investigations to different disease states, as already done within the framework of the MAGYAR-Path Study [7,8]. Furthermore, exploring these associations using other cardiovascular imaging techniques, such as magnetic resonance imaging, would be worthwhile.

**Limitation section:** The most important limitations that arose during the study are as follows:

- A limitation of this study is the lack of a formal a priori power analysis to determine the ideal sample size. While the total cohort included 109 participants, the subgroup analyses presented in Table 2, Table 3 and Table 4 involved small group sizes (n < 20 in the upper and lower percentiles). This relatively small sample size may have limited the statistical power to detect subtle associations between the tested parameters, potentially leading to Type II errors. Despite this, baseline characteristics showed a relative homogeneity across groups. However, our findings should be considered exploratory, and further studies with larger, pre-calculated sample sizes are required to confirm the absence of these associations.
- Although the study population consisted of individuals considered clinically healthy, an absolute confirmation of health status cannot be guaranteed, as additional exclusionary diagnostic tests would have been required.
- The image quality of 3DSTE remains inferior to that of conventional 2D echocardiography, largely due to its lower temporal and spatial resolution and the larger transducer size, which together limit its applicability [11,12,13,14,15].
- Parameters obtained from other imaging modalities were not included or compared within the scope of this study.
- It was not our purpose to analyze AVA dimensions throughout the cardiac cycle or to relate these parameters to LV volumes [6]. This may instead be a topic for future investigations.
- The study did not aim to compare AAPSE values derived from M-mode echocardiography with those obtained using 3DSTE.
- Although 3DSTE allows for a broad range of additional assessments—such as chamber quantification or an evaluation of the atrioventricular valvular annuli—these analyses fell outside the objectives of the current investigation [16,18,19,20].
- Indexation of LV volumes would have helped with more accurate comparisons between subgroups.
- Despite the well-recognized anatomical and functional relationship between the AVA and the aorta, this relationship was not evaluated in the present study.

## 5. Conclusions

LV volumes and the spatial excursion of the AVA represented by the AAPSE are not associated in healthy adults, as measured simultaneously using 3DSTE.

## Figures and Tables

**Table 1:** Clinical and two-dimensional echocardiographic data.

Data	Measures
**Clinical data**	
**n**	109
**Mean age (years)**	35.1 ± 12.0
**Males (%)**	68 (62)
**Systolic blood pressure (mmHg)**	123 ± 4
**Diastolic blood pressure (mmHg)**	82 ± 3
**Heart rate (1/s)**	73 ± 3
**Height (cm)**	73.3 ± 16.2
**Weight (kg)**	175.2 ± 11.3
**Two-dimensional echocardiographic data**	
**LA diameter (mm)**	37.5 ± 3.5
**LV end-diastolic diameter (mm)**	48.1 ± 3.6
**LV end-systolic diameter (mm)**	32.3 ± 2.9
**LV end-diastolic volume (mL)**	107.3 ± 21.9
**LV end-systolic volume (mL)**	38.2 ± 9.2
**Interventricular septum (mm)**	9.2 ± 1.3
**LV posterior wall (mm)**	9.4 ± 1.3
**LV ejection fraction (%)**	64.5 ± 3.6
**Early diastolic mitral inflow velocity—E (cm/s)**	77.9 ± 15.5
**Late diastolic mitral inflow velocity—A (cm/s)**	59.0 ± 14.1

**Abbreviations:** LA = left atrial, LV = left ventricular.

**Table 2:** Aortic valve annular dimensions and left ventricular volumes in different aortic valve annular plane systolic excursion groups.

	AAPSE ≤ 0.86 cm (n = 17)	0.86 cm < AAPSE < 1.46 cm (n = 74)	1.46 cm ≤ AAPSE (n = 18)
**AAPSE (mm)**	0.71 ± 0.10	1.16 ± 0.16 *	1.63 ± 0.18 *^†^
**LV-EDV (mL)**	82.6 ± 22.2	88.3 ± 19.1	83.5 ± 29.2
**LV-ESV (mL)**	35.2 ± 11.9	37.3 ± 10.0	35.4 ± 11.3
**LV-EF (%)**	57.9 ± 5.4	57.4 ± 5.6	59.8 ± 6.1
**LV mass (g)**	168.0 ± 29.9	165.4 ± 30.4	159.4 ± 38.1

**Abbreviations:** AAPSE = aortic valve annular plane systolic excursion; LV = left ventricular, EDV = end-diastolic volume, ESV = end-systolic volume, EF = ejection fraction. * *p* < 0.05 vs. AAPSE ≤ 0.86 cm; ^†^ *p* < 0.05 vs. 0.86 cm < AAPSE < 1.46 cm.

**Table 3:** Aortic valve plane systolic excursion and left ventricular volumes in different left ventricular end-diastolic volume groups.

	LV-EDV ≤ 66.1 mL (n = 13)	66.1 mL < Global LV-EDV < 107.7 mL (n = 84)	107.7 mL ≤ Global LV-EDV (n = 12)
**AAPSE (mm)**	1.07 ± 0.28	1.18 ± 0.31	1.17 ± 0.29
**LV-EDV (mL)**	56.5 ± 5.3	84.9 ± 11.7 *	126.5 ± 16.7 *^†^
**LV-ESV (mL)**	24.5 ± 5.0	35.5 ± 7.1 *	56.1 ± 8.8 *^†^
**LV-EF (%)**	57.8 ± 6.1	58.2 ± 5.8	55.6 ± 3.9
**LV mass (g)**	131.8 ± 24.5	163.2 ± 26.7 *	208.5 ± 25.3 *^†^

**Abbreviations:** AAPSE = aortic valve annular plane systolic excursion, LV = left ventricular, EDV = end-diastolic volume, ESV = end-systolic volume, EF = ejection fraction. * *p* < 0.05 vs. LV-EDV ≤ 66.1 mL; ^†^ *p* < 0.05 vs. 66.1 mL < LV-EDV < 107.7 mL.

**Table 4:** Aortic valve plane systolic excursion and left ventricular end-systolic volumes in different left ventricular end-systolic volume groups.

	LV-ESV ≤ 26.1 mL (n = 16)	26.1 mL < Global LV-ESV < 47.3 mL (n = 75)	47.3 mL ≤ Global LV-ESV (n = 18)
**AAPSE (mm)**	1.12 ± 0.30	1.15 ± 0.29	1.24 ± 0.32
**LV-EDV (mL)**	66.3 ± 14.2	82.4 ± 14.8 *	119.7 ± 17.5 *^†^
**LV-ESV (mL)**	22.6 ± 2.7	35.6 ± 5.7 *	53.7 ± 6.2 *^†^
**LV-EF (%)**	65.0 ± 5.6	57.1 ± 4.9 *	54.9 ± 3.4 *
**LV mass (g)**	138.2 ± 27.8	163.1 ± 26.7 *	195.5 ± 30.1 *^†^

Abbreviations: * *p* < 0.05 vs. LV-ESV ≤ 26.1 mL; ^†^ *p* < 0.05 vs. 26.1 mL < LV-ESV < 47.3 mL.

**Table 5:** Intra- and interobserver variability for aortic valve annular plane systolic excursion and left ventricular volumes as assessed simultaneously using three-dimensional speckle-tracking echocardiography.

	Intraobserver Agreement		Interobserver Agreement	
	Mean ± 2SD Difference in Values Obtained by Two Measurements of the Same Observer	ICC Between Measurements of the Same Observer	Mean ± 2SD Difference in Values Obtained by Two Observers	ICC Between Independent Measurements of Two Observers
**AAPSE (cm)**	−0.03 ± 0.17	0.92 (*p* < 0.01)	−0.03 ± 0.15	0.92 (*p* < 0.01)
**LV-EDV (mL)**	1.6 ± 5.9	0.91 (*p* < 0.01)	1.6 ± 5.0	0.90 (*p* < 0.01)
**LV-ESV (mL)**	0.7 ± 4.4	0.91 (*p* < 0.01)	0.9 ± 4.1	0.91 (*p* < 0.01)

**Abbreviations:** ICC = intraclass correlation coefficient, AAPSE = aortic valve annular plane systolic excursion, LV = left ventricular, EDV = end-diastolic volume, ESV = end-systolic volume.

## Data Availability

The original contributions presented in the study are included in the article, further inquiries can be directed to the corresponding authors.
